# Nanometer size silicon particles for hyperpolarized MRI

**DOI:** 10.1038/s41598-017-08709-0

**Published:** 2017-08-11

**Authors:** Grzegorz Kwiatkowski, Fabian Jähnig, Jonas Steinhauser, Patrick Wespi, Matthias Ernst, Sebastian Kozerke

**Affiliations:** 1 Institute for Biomedical Engineering, University and ETH Zurich, Zurich, Switzerland; 2 Laboratory of Physical Chemistry, ETH Zurich, Zurich, Switzerland

## Abstract

Hyperpolarized silicon particles have been shown to exhibit long spin-lattice relaxation times at room temperature, making them interesting as novel MRI probes. Demonstrations of hyperpolarized silicon particle imaging have focused on large micron size particles (average particle size (APS) = 2.2 μm) as they have, to date, demonstrated much larger polarizations than nanoparticles. We show that also much smaller silicon-29 particles (APS = 55 ± 12 nm) can be hyperpolarized with superior properties. A maximum polarization of 12.6% in the solid state is reported with a spin-lattice relaxation time of 42 min at room temperature thereby opening a new window for MRI applications.

## Introduction

Theranostics application of micro- and nano-objects is an emerging field in biomedical sciences. In particular, silicon and its derivatives have been employed to constitute a targeted, site-specific platform for possible drug delivery and *in-vivo* imaging^1^. Its relatively low toxicity^2^ and simple surface chemistry^3^ imply that silicon can be incorporated as a contrast agent in various imaging modalities, including fluorescence^4, 5^ or radioisotope (PET)^6^ methods.

For Magnetic Resonance Imaging (MRI) applications, silicon based contrast agents have been obtained by incorporating transition metal ions into a particle’s body^7, 8^ or by attaching them on its surface^9^. This results in a shortening of the nuclear spin-lattice relaxation time (*T*_1_) of nearby tissue protons and hence signal amplification in *T*_1_-weighted proton imaging. A direct detection of silicon signals with MRI is however not possible due to the intrinsically low sensitivity of ^29^Si nuclei, which leads to impractically long acquisition times.

An approach to overcome this limitation is by a use of hyperpolarization^10^ - a family of techniques in which the nuclear polarization is temporarily increased beyond its equilibrium value, usually by a factor of 10^3^–10^5^. Different physical mechanisms can be employed to increase the nuclear polarization including optical spin-exchange pumping^11^, hydrogenation reaction^12^ or direct transfer of spin angular momentum from electron spins (e.g. in stable radicals). The latter mechanism, referred to as Dynamic Nuclear Polarization (DNP), has received particular attention^13^. Combining low temperature DNP of ^13^C labeled metabolites with a rapid dissolution of the sample^14^ allows mapping its metabolic conversion *in-vivo*, in both spectral and spatial dimensions^15^. However, the time in which this large polarization can be used is limited by the nuclear spin-lattice relaxation time of the spin species. In the case of ^13^C nuclei, the imaging window span lasts around 60–120 s^16^. This time, although short, allows rapid enzymatic reactions, e.g. anaerobic metabolism, to be studied, which can then be further used to characterize pathology of tissue^17, 18^.

If, however, slow biological processes such as protein-cell binding, intragastric transit or tissue perfusion and exchange are of interest, much longer life times of the hyperpolarized signal are required. To this end, there have been efforts to extend the relaxation times of ^1^H or ^13^C nuclei beyond the *T*_1_ limit by storing the magnetization in “long-lived” states^19^ which are, for symmetry reasons, protected from many relaxation mechanisms. While such an approach works well on some model systems in the laboratory^20, 21^, *in-vivo* applications of such concepts have not been demonstrated so far. Accordingly, alternative hyperpolarized MRI contrast agents, characterized by a long lifetime and *in-vivo* applicability, are desired.

One such potential candidate is silicon in the form of micro and nanoparticles^22^. Recent results from experiments with micrometer size ^29^Si particles have shown a lifetime of the hyperpolarized signal on the order of tens of minutes^23–25^, exceeding that of any other ^13^C based probe reported so far. Therefore, by combining the enhancement of ^29^Si polarization using DNP together with the long relaxation properties of crystalline silicon, a new, positive, background-free MRI contrast can be obtained. The proof-of-principle studies showed that silicon microparticles can be administrated via either intragastric (I.G.), intraperitoneal (I.P.) or intravenous (I.V.) routes without loss of any of the long-lived properties^26^. However, the reported studies were only able to incorporate large, micrometer-size particles that are of limited biomedical application due to their restricted distribution within the tissues. Data using nanometer-size particles revealed rather poor performance, exhibiting short *T*_1_ relaxation time and/or small DNP enhancement, which are not sufficient for *in-vivo* MRI applications^27, 28^.

The objective of the present work is to introduce nanometer-size silicon particles obtained with laser ablation and demonstrate their suitability for MRI upon hyperpolarization.

## Results

### Silicon nanoparticles

**Figure 1:**
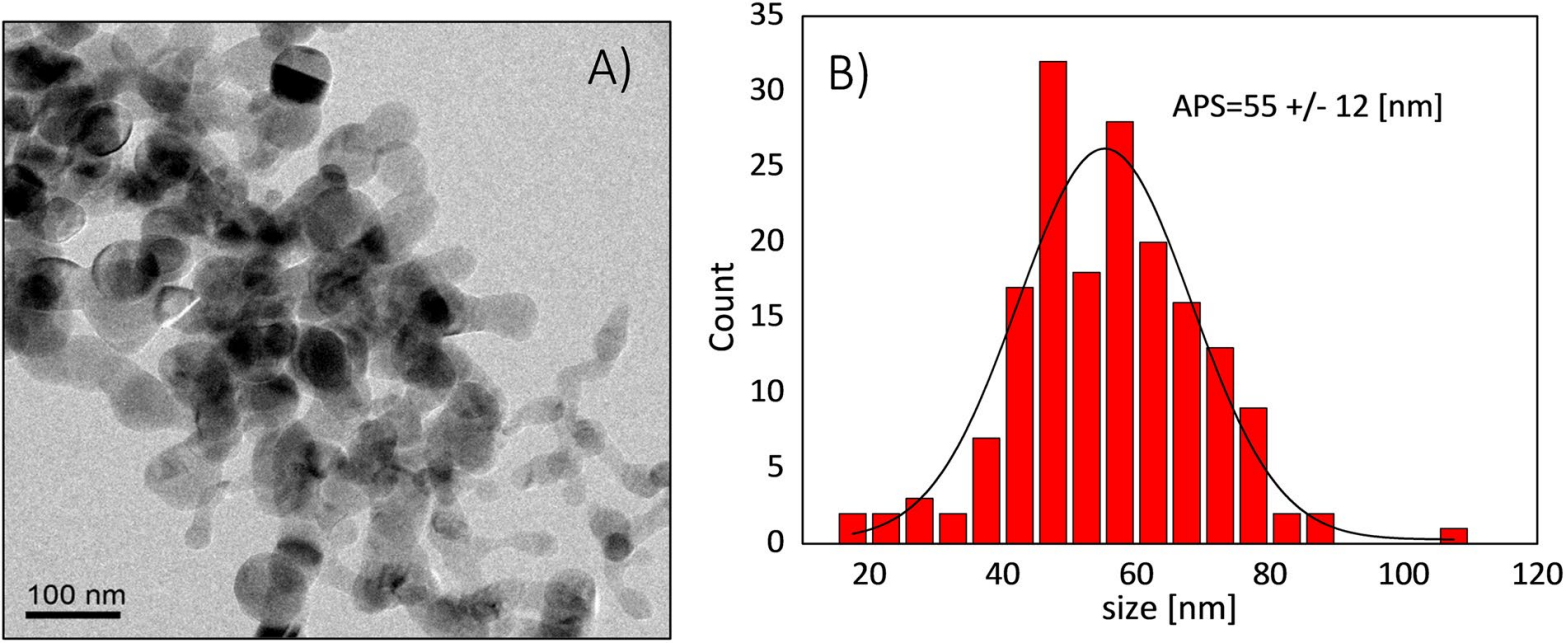
(**A**) TEM image of dry silicon nanopowder. (**B**) Size distribution obtained from 10 subsequent TEM images.

Transmission electron microscopy (TEM) imaging of the examined silicon powder sample yielded an average particle size (APS) of 55 ± 12 nm (Fig. 1). Each particle is composed of an elemental silicon bulk (predominantly in a crystalline phase) and an oxidized layer on the surface. The naturally occurring defects^29, 30^ (dangling bonds) between crystalline silicon and silicon dioxide serve as a source of polarization that can be transferred to ^29^Si nuclei by dynamic nuclear polarization (DNP) using microwave irradiation (see Supplementary Information S1). Accordingly, no doping of the sample with exogenous radicals as well as glassing solvents are required.

### Hyperpolarization of silicon nanoparticles

**Figure 2:**
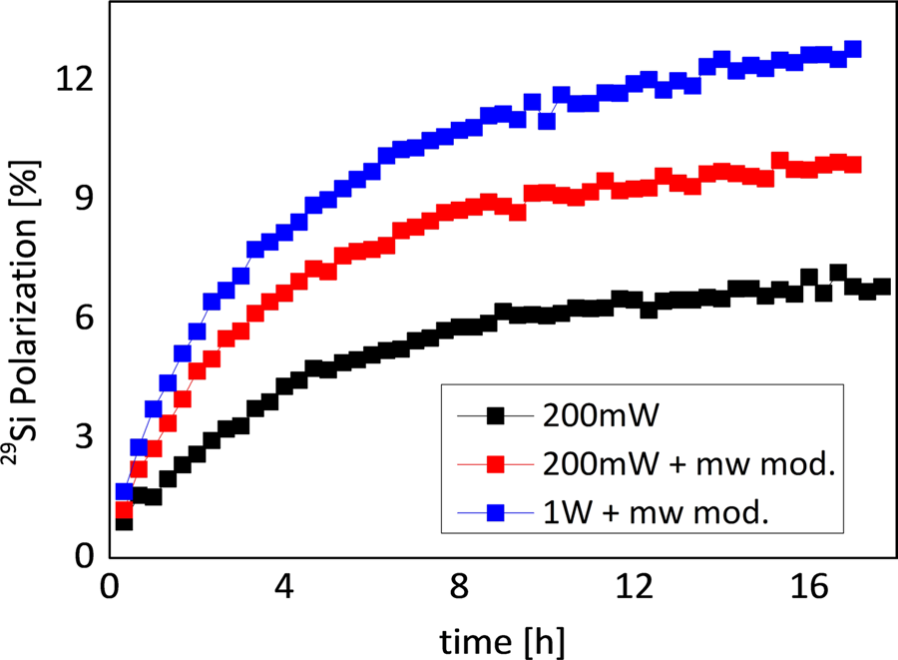
Build-up curves of ^29^Si nuclear polarization using 200 mW microwave power (black), additional microwave modulation (red) and with a 1 W power amplifier and microwave modulation (blue). Maximum achievable polarization was 12.6%.

The ^29^Si nuclear polarization could be significantly enhanced using dynamic nuclear polarization. The time constant of the polarization build-up was ~4.5 h and did not depend on the microwave power. Such a long time constant for the build-up of the bulk polarization is attributed to slow spin diffusion from the polarized surface atoms to the bulk ^29^Si in the interior of a particle. Spin diffusion is limited by the relatively low concentration of ^29^Si nuclei at natural abundance (4.7%). The maximum achievable polarization for dry material could be substantially enhanced by employing a frequency-modulation scheme^31, 32^ and a 1 W power amplifier (Fig. 2), resulting in a final polarization level of 12.6%.

**Figure 3:**
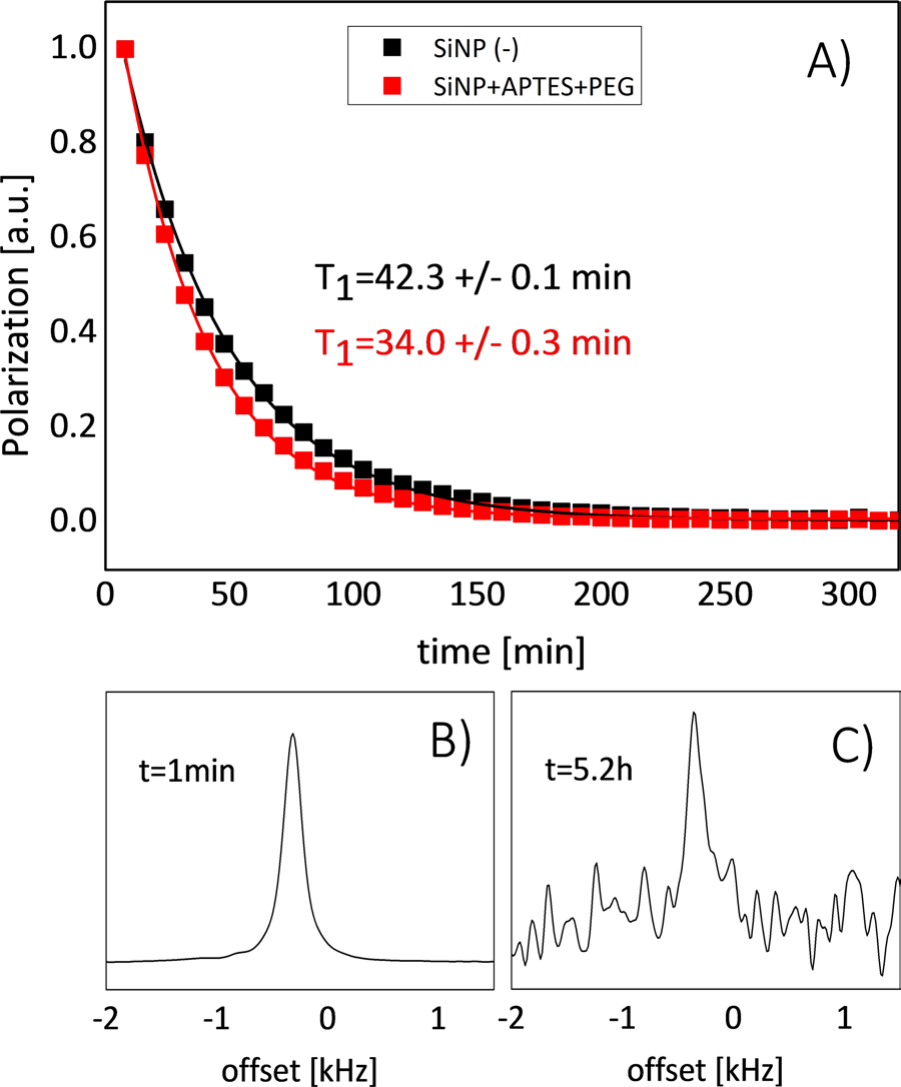
(**A**) Comparison of *T*_1_ relaxation between pure (black) and PEG functionalized (red) silicon nanoparticles. The Fourier-transformed FID signal acquired at *t = *1 min (**B**) and *t* = 5.2 h (**C**) at room temperature using a 9.4 T imaging system.

The silicon nanoparticles exhibit a long *T*_1_ relaxation time of 42.3 ± 0.1 min (Fig. 3A). Such a long depolarization time enabled the observation of ^29^Si NMR signal (SNR > 3) more than 5 h after transfer to the imaging system (Fig. 3B). The presence of PEG polymer on the surface of the nanoparticles resulted in shortening of *T*_1_ relaxation times at room temperature from 42.3 ± 0.1 min to 34.0 ± 0.3 min (Fig. 3A), while no change in the polarization level was observed.

### MR imaging

**Figure 4:**
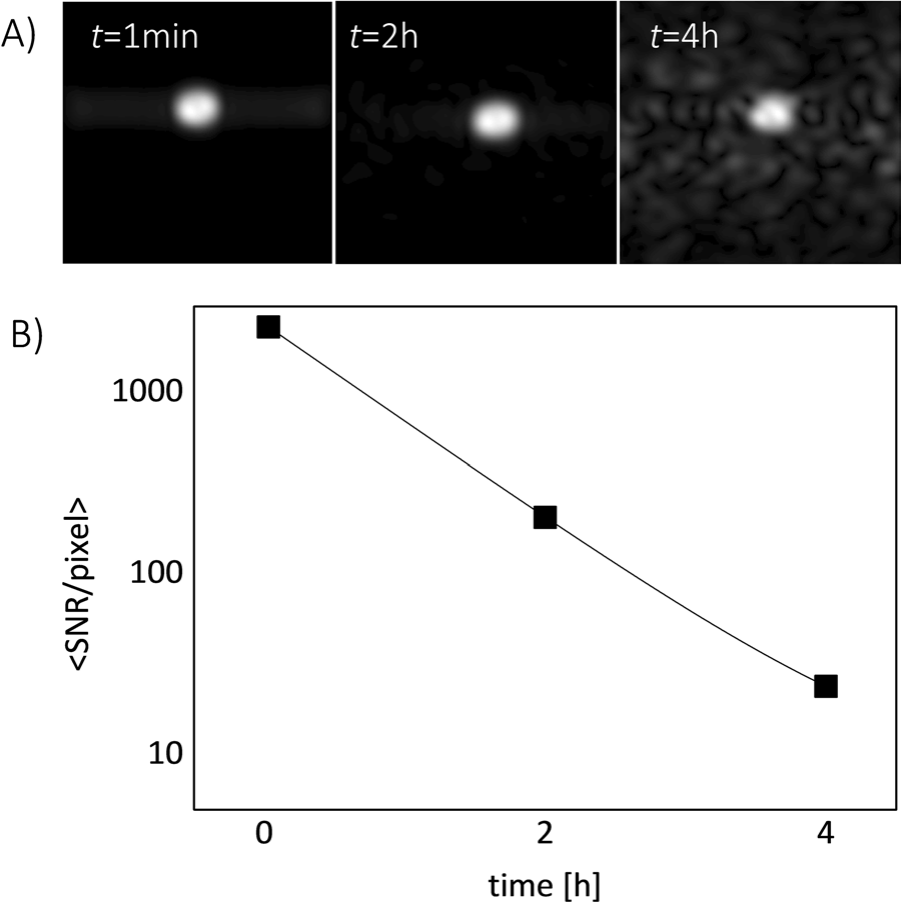
(**A**) Three consecutive images of a dry powder obtained after 1 min, 2 h and 4 h after the transfer to the imaging system. Each sample was polarized for 24 h before imaging. (**B**) Decay of average SNR/pixel with time after transferring the sample to the imaging system.

High quality images were obtained by exploiting the long dephasing time of silicon nuclei which has been shown to occur under periodic excitation^33^ (Fig. 4). Due to relatively small nuclear dipolar interaction, the spin echo can be fully re-phased with a standard CPMG sequence, increasing decoherence (*T*_2_) time by a factor of more than a hundred^34^.

The sensitivity of the recorded signal was probed as a function of the waiting time after sample transfer to the imaging system to test the time available for the nanoparticles to be distributed within the tissue. As expected, the mean SNR/pixel value decayed with time given by the *T*_1_ relaxation time (Fig. 4B) but nevertheless providing sufficient SNR for imaging after four hours.

**Figure 5:**
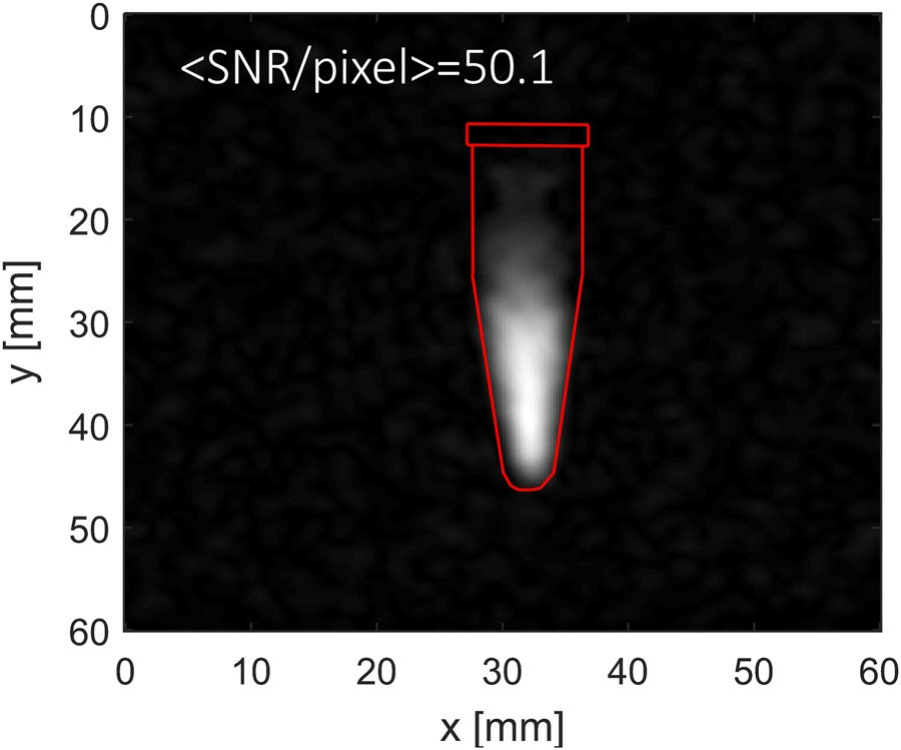
Image of 30 mg of functionalized silicon nanoparticles dispersed in 500 μl of water. The sample was polarized for *t* = 8 h. The image was taken immediately after transfer of the sample to the imaging system. The red overlay indicates the outline of the water vial into which the silicon was dispersed.

Imaging of silicon in PBS buffer resulted in images of good quality, with the shape of the phantom clearly visible (Fig. 5) without any thresholding or extensive post-processing. The gradient of the signal intensity over the sample is due to the majority of the emulsion aggregating in the lower part of the vial.

## Discussion

Early reports of hyperpolarized silicon particles demonstrated MRI feasibility of large particles (APS = 2.2 μm, obtained with ball-milling of a silicon ingot), while nanometer-size material showed relatively poor performance with *T*_1_ relaxation on the order of a few minutes^27, 28^. MRI has not been possible with the nanometer-size particles due to intrinsically low achievable polarization levels^35^. In contrast, the nanoparticles used in the present study show superior quality in both relaxation time (42 min for pure particles) and polarization level (12.6%), allowing high quality images to be obtained up to four hours after transportation to the imaging system. In this respect, the data presented here significantly outperforms the initial results in ref. 26, where the image could only be acquired after up to 1.5 h following the transfer.

A further significant increase in the achievable polarization level is expected by lowering the temperature during the DNP process. Using a dedicated long-run helium bath or helium-free cryostat will allow for DNP at significantly lower temperature (<2 K) for virtually any time period, boosting the obtainable ^29^Si polarization. Moreover, conducting the DNP process at higher magnetic fields is expected to additionally contribute to higher final nuclear polarization. Currently, the limited availability of high-power microwave sources operating at frequencies above 100 GHz presents the major obstacle.

Large particles, especially in the micrometer size range, are difficult to administer and handle *in-vivo*. Issues include blockage of nearby veins and arteries or sedimentation at the site of injection resulting in poor bio-distribution and -compatibility. The small size of the nanoparticles presented here should facilitate greater *in vivo* mobility^5^.

A particular advantage of using silicon as a contrast agent is its versatility of surface chemistry^36^. As it has been shown, the attachment of functional organic molecules on the surface of particles does not significantly reduce any of the desired NMR properties. Further development will be focused on the applications of a specific surface functionalization to produce targeted probes.

The discrepancy between the mean SNR/pixel obtained for dry sample and nanoparticles dispersed in a solution arises not only from the dilution factor and reduced polarization time but also due to an additional polarization loss that occurs when the material is dispersed in a solvent (Fig. S5). A potential cause of this behavior could be due to a temporary increase in relaxation caused by rapid boost in rotational motion of silicon nanoparticles. Nonetheless, this process needs further investigation. Application of a dedicated instrument that allows for rapid flushing of the dry material will reduce the time of dissolution and is hence expected to preserve nuclear polarization better in the future.

## Conclusion

In this work, nanometer size silicon nanoparticles with favorable relaxation properties have been polarized beyond previous limits and imaged successfully demonstrating their potential as contrast agents for MRI applications.

## Materials and Methods

### Silicon nanoparticles

Silicon nano-powder, synthesized from a gas phase using a laser-assisted technique, (US Nano Research, Houston, TX, USA) was used without further modification. The silicon particles are characterized by an elemental purity of >99.99% and ^29^Si natural abundance of 4.7%. The advantages of using silicon particles obtained with a laser-assisted technique include narrow-size distribution, high purity and relatively large crystalline size, especially compared to ball-milled silicon^37^.

### Hyperpolarization

A home-built polarizer operating at *B*_0_ = 3.4 T at a temperature of ~3.5 K was used^38^. The sample was composed of 100 mg tightly packed powder enclosed in a polytetrafluoroethylene (PTFE) cup. To enhance polarization, the microwave field was frequency-modulated^31, 32^ using a symmetric ramp function with a frequency *ν* = 3 kHz and a bandwidth of 150 MHz. An additional boost in microwave power was achieved by feeding the output of the microwave source (ELVA-1 VCOM-10/94/200-DD, max power 200 mW) into a 95 GHz 1 W power amplifier (QuinStar, Torrance, USA). For measurements in the solid state, a probe with a solenoid coil wound around the sample cup was used. The microwave field was delivered by direct irradiation from the waveguide elbow. The absolute value of polarization in the solid state was obtained by comparing the integrated signal intensity of the hyperpolarized sample with the thermal-equilibrium signal of 100 mg fully labelled silicon at 295 K (99% of ^29^Si, Isofelx, Moscow, Russia).

### MR Imaging

For measurements of dissolved samples in the imaging system, a probe with a custom-design, quasi-cavity structure was used to ensure a homogenous microwave *B*_1_ field profile and hence even distribution of polarization throughout the sample^38^. All imaging experiments were conducted following the same protocol. After 24 h of continuous polarization, the samples were taken out of the polarizer and immediately transferred to the face of a horizontal 9.4 T imaging system (Bruker BioSpin, Ettlingen, Germany). The sample was placed in a home-built, semi half-saddle surface coil (operating at Larmor frequency *ν* = 79 MHz, 44 × 44 mm^2^ size). Silicon imaging was performed using the Rapid Acquisition with Refocused Echoes (RARE) sequence^39^. The dry samples were imaged with a 32 × 32 matrix and 55% partial Fourier, while the dispersed samples were imaged with a 64 × 64 matrix and 71% partial Fourier. A standard RARE sequence provided in ParaVision^®^ 6.0 was used with the following parameters: TE/TR = 2.75/51.75 ms, excitation pulse length = 230 μs, refocusing pulse length = 350 μs, FOV = 60 × 60 mm, slice thickness = 15 mm. For post-processing the raw data were imported into an in-house developed Matlab (The Mathworks, Natick, USA) code. The frequency domain data has been apodized with a Hamming window and zero filled to a 512 × 512 matrix. Silicon spectroscopy was recorded with a train of low-angle (*θ* = 10°, TR = 8 min) pulses over an 8 h period.

### Surface modification

To improve the biodistribution and biocompatibility of the nanoparticles, the surface of silicon nanoparticles was functionalized with polyethylene glycol^40^ (see Supplementary Information S2 for details). As a proof-of-principle for *in-vivo* applications, the surface-modified silicon nanoparticles were imaged after dispersion in a phosphate-buffered saline (PBS). The amount of material was reduced to 30 mg (60 mg/mL), and in addition, the polarization time was also reduced to 8 h, which should allow for two-shift operation of the DNP polarizer.

### Data Availability

All data generated or analyzed during this study are included in this published article (and its Supplementary Information files).

## Electronic supplementary material

Electronic Supplementary Information
