# Supplementary material for: Nanometer size silicon particles for hyperpolarized MRI
Source: Sci Rep. 2017 Aug 11;7:7946. doi: 10.1038/s41598-017-08709-0 (PMC5554256; doi:10.1038/s41598-017-08709-0)
Supplement: Supplementary file 1 — Electronic Supplementary Information [file 41598_2017_8709_MOESM1_ESM.pdf]

Electronic Supplementary Information (ESI)

Nanometer size silicon particles for hyperpolarized MRI

Grzegorz Kwiatkowski[1,2], Fabian Jähnig[2], Jonas Steinhauser[1], Patrick Wespi[1], Matthias Ernst[2],

Sebastian Kozerke[1]

[1]Institute for Biomedical Engineering, University and ETH Zurich, Switzerland;

[2]Laboratory of Physical Chemistry, ETH Zurich, Switzerland

1. Material characterization

The silicon powder was used as obtained from the manufacturer and stored in an air-tight container. No special precautions were taken to prevent air exposure. At the same time, no degradation or change in any of the material properties described in this communication were observed over the period of 10 months.

The average crystalline size and crystal-to-amorphous phase ratio were measured using a Bruker ADVANCE D8 XRD spectrometer. All analysis was made using the manufacturer's dedicated software.

The origin of the paramagnetic properties of the nanoparticles was confirmed with EPR spectroscopy. A CW spectrum was recorded at room temperature using a Bruker ElexSys E580 spectrometer operating at X-band frequency.

Both XRD and EPR spectra confirmed high purity and homogeneity of the silicon particles (Figure S1). Lack of low-angle dispersion in the XRD spectrum confirmed predominantly crystalline structure of silicon. A single resonance was observed in the EPR spectrum at a g-value of around ~2.006 which is characteristic for Pb paramagnetic centers [1,2].

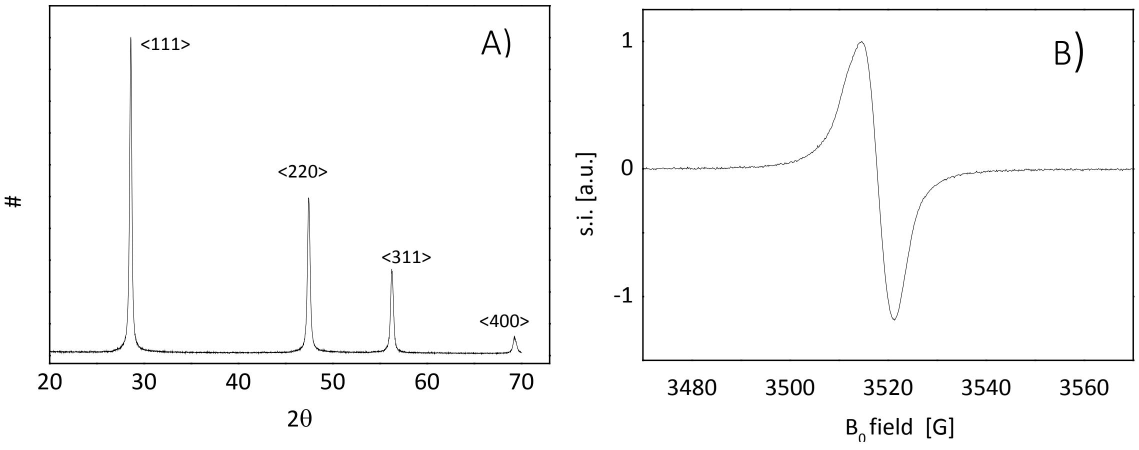

Figure S1. A) X-ray diffraction spectroscopy spectrum of powder silicon nanoparticles. The crystal plane assignment was performed according to a well-known silicon standard. B) EPR spectrum recorded in CW dispersion mode.

2. Particle surface functionalization procedure

All intermediate chemicals were used as purchased. Ethanol (puris) and (3-Aminopropyl)-triethoxysilane were sourced from Sigma-Aldrich (Switzerland). An ester of polyethylene glycol in a form of NHS-dPEG$_4$-(m-PEG$_{12}$)$_3$-ester was obtained from Quanta Biodesign (Ohio, USA). In order to obtain results comparable with previous communications, the same protocol as described in [3] was followed.

Amination

In short, 100 mg of pure silicon nanoparticles were dispersed in 22.5 ml of acidified ethanol (pH~3.5, adjusted with HCl) and sonicated for 10 min. Following that, 0.79 ml of (3-aminopropyl)triethoxysilane (APTES) was added and the solution was stirred for 20 h at 700 rpm on a magnetic stirrer plate. The unreacted APTES was removed but repeated washing with ethanol buffer. Surface amination was confirmed with a fluorescamine test.

PEG-ylation

100 mg of aminated silicon nanoparticles was dispersed in 1 ml of ethanol and heated to 50° C on a magnetic stirring plate. Next, 100 mg of PEG ester was added and stirred at 700 rpm for 24 h at room temperature.

The increased solubility was confirmed by dispersing both pure and surface-modified material in 1 ml of PBS buffer. Following 10 min of sonication, the solutions were left for 30 min and visually inspected for sedimentation. The pure nanoparticles were found to precipitate out of the solution and fully sedimented, while the functionalized material was still homogenously dispersed (Figure S3-A). In addition, each step of the surface modification was confirmed with FT-IR spectroscopy, using a Bruker Alpha FT-IR spectrometer working in attenuated total reflection sampling mode (Figure S3-B).

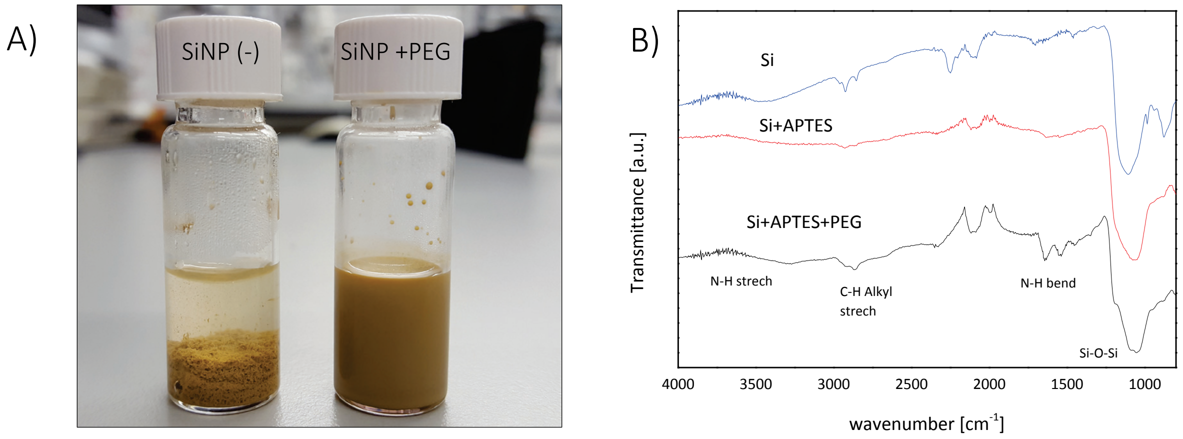

Figure S2. A) Photography of pure (SiNP(-)) and functionalized (SiNP+PEG) nanoparticles 30min post dispersion in PBS buffer. B) Transmission mode ATR spectra of pure silicon (Si), amine functionalized (Si+APTES) and polyethylene glycol functionalized (Si+APTES+PEG) nanoparticles. The spectra were shifted in y-direction for better readability.

Size distribution measurement

The total radius of the nanoparticles is expected to increase upon functionalization. The hydrodynamic radius of pure and functionalized material was confirmed by employing dynamic light scattering (DLS). All measurements were done using a Zetasizer Nano Z (Malver) instrument. Less than 1 mg of material was dispersed in 5 ml of distilled water. The measurements were conducted at room temperature with triple repetitions. The size distribution was modeled with a Gaussian distribution (Figure S3).

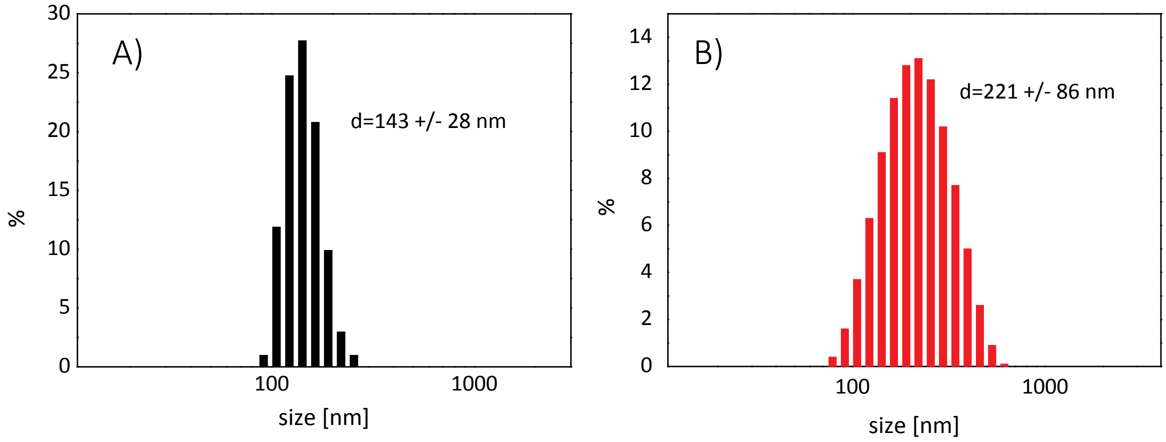

Figure S3. Size distribution of a hydrodynamic radius of pure (A) and functionalized (B) nanoparticles in distilled water.

Although a significant increase in particles radius was observed (by almost a factor of 2), the average diameter of the nanoparticles is still within an acceptable range for theranostics applications [4] (Table S1). Further improvement of the functionalization protocol, especially of the amination time [5–7], in addition to application of high power sonication prior to measurements is expected to minimize effective size of functionalized particles. Furthermore application of zeta potential measurements could help to elucidate detrimental factors affecting particle aggregation.

Abraxane	Albumin-bound Paclitaxel	80-120nm
Feridex®	Dextran-coated SPIOs	120-180nm
Doxil®	Liposomal doxorubicin	80-120nm

Table S1. List of FDA approved and clinically used pharmaceutical products based on nanoparticles with their respective size.

3. Effect of particle surface functionalization

Build-up of ^{29}Si polarization was compared between pure and functionalized material. No effect on build-up time as well as maximum achievable polarization was found (Figure S4-A). In addition, the NMR spectra for both samples were recorded at a 9.4 T imaging system (corresponding to the Larmor frequency ν = 79 MHz). No line broadening due to close proximity of PEG protons was observed (Figure S4-B), proving that functionalization can be performed without any loss in DNP/NMR properties of silicon nanoparticles.

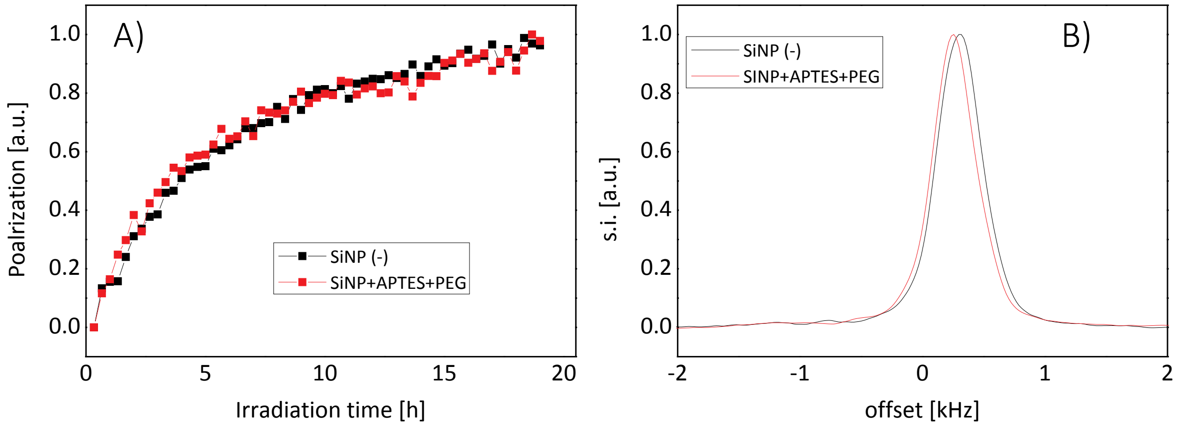

Figure S4. A) Comparison of polarization build-up between pure (black) and functionalized (read) nanoparticles. B) Respective NMR spectra recorded at f=79MHz and at room temperature of pure (black) and functionalized (read) nanoparticles immediately after transfer from the polarizing magnet.

4. Loss of ^{29}Si polarization upon dispersion in a solvent

The polarization step was performed on dry silicon powder. Prior to injection, the nanoparticles were dispersed in a solution by manual agitation with a syringe. It has been observed that upon dispersion a substantial loss of ^{29}Si polarization occurs.

The material was polarized as for previous experiments and transferred to the imaging system. A single free induction decay (FID) signal was acquired with a θ = 20° pulse. Immediately after the nanoparticles were dispersed in 650 µl of distilled water, a second FID was acquired. The dispersion procedure was carried out at the face of the magnet to avoid any additional relaxation at low magnetic field. As shown in Fig. S5, up to 50% of the initial polarization is lost upon dispersion. At the same time, no change in the line-shape is observed which would be an indication of a rapid motion of silicon nanoparticles in the solution. Such a large loss in polarization could be due to faster relaxation of the nanoparticles caused by a

temporary boost in rotational motion. As a result, the highly polarized ^{29}Si nuclei that are closed to surface defects relax instantaneously, leaving only the magnetization from the interior of a particle. Similar results was observed by Cassidy et al. [8] where up to 90% of the initial polarization was lost.

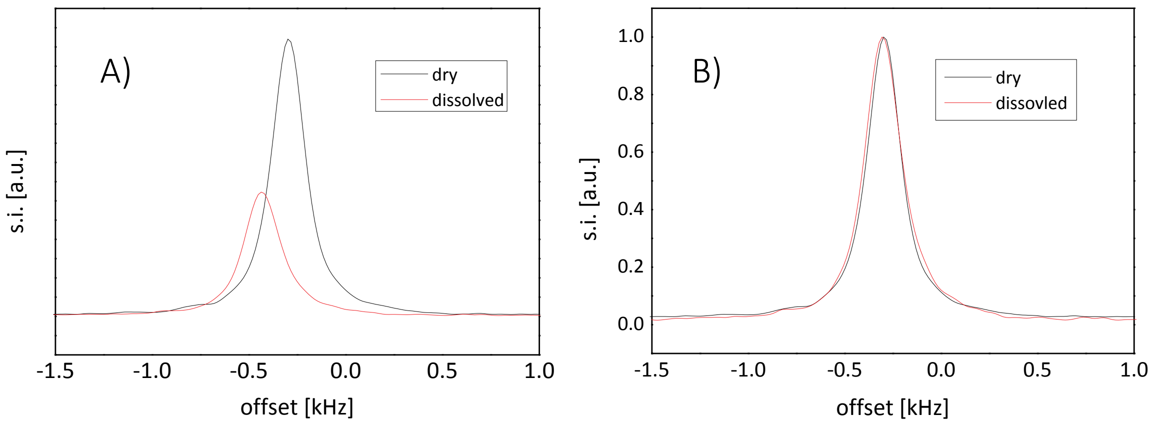

Figure S5. Signal intensity (A) and line shape comparison (B) for dry (black) and dispersed in a solution (red) silicon nanoparticles. The shift in frequency in (A) is addressed to a misalignment of a sample cradled when shuffled back after dispersion.

References

1. Poindexter, E. H. & Caplan, P. J. Characterization of Si/SiO2 Interface Defects by Electron Spin Resonance. *Prog. Surf. Sci.* **14,** 201–294 (1983).
2. Teutloff, C. Dangling bonds in amorphous silicon investigated by Multifrequency EPR. (2012). doi:10.1016/j.jnoncrysol.2011.12.105
3. Aptekar, J. W. *et al.* Hyperpolarized Long-T1 Silicon Nanoparticles for Magnetic Resonance Imaging. 1–5 (2009). doi:10.1021/nn900996p
4. Ehlerding, E. B., Chen, F. & Cai, W. Biodegradable and Renal Clearable Inorganic Nanoparticles. *Adv. Sci.* **3,** 1500223 (2016).
5. Liu, Y., Li, Y., Li, X. M. & He, T. Kinetics of (3-aminopropyl)triethoxylsilane (aptes) silanization of superparamagnetic iron oxide nanoparticles. *Langmuir* **29,** 15275–15282 (2013).
6. Howarter, J. A. & Youngblood, J. P. Optimization of silica silanization by 3-aminopropyltriethoxysilane. *Langmuir* **22,** 11142–11147 (2006).
7. Acres, R. G. *et al.* Molecular structure of 3-aminopropyltriethoxysilane layers formed on silanol-terminated silicon surfaces. *J. Phys. Chem. C* **116,** 6289–6297 (2012).
8. Cassidy, M. C., Chan, H. R., Ross, B. D., Bhattacharya, P. K. & Marcus, C. M. In vivo magnetic resonance imaging of hyperpolarized silicon particles. *Nat. Nanotechnol.* **8,** 363–368 (2013).
